# A human β-III-spectrin spinocerebellar ataxia type 5 mutation causes high-affinity F-actin binding

**DOI:** 10.1038/srep21375

**Published:** 2016-02-17

**Authors:** Adam W. Avery, Jonathan Crain, David D. Thomas, Thomas S. Hays

**Affiliations:** 1 Department of Genetics, Cell Biology and Development, University of Minnesota, Minneapolis, MN 55455; 2 Department of Biochemistry, Molecular Biology, and Biophysics, University of Minnesota, Minneapolis, MN 55455

## Abstract

Spinocerebellar ataxia type 5 (SCA5) is a human neurodegenerative disease that stems from mutations in the *SPTBN2* gene encoding the protein β-III-spectrin. Here we investigated the molecular consequence of a SCA5 missense mutation that results in a L253P substitution in the actin-binding domain (ABD) of β-III-spectrin. We report that the L253P substitution in the isolated β-III-spectrin ABD causes strikingly high F-actin binding affinity (Kd = 75.5 nM) compared to the weak F-actin binding affinity of the wild-type ABD (Kd = 75.8 μM). The mutation also causes decreased thermal stability (Tm = 44.6 °C vs 59.5 °C). Structural analyses indicate that leucine 253 is in a loop at the interface of the tandem calponin homology (CH) domains comprising the ABD. Leucine 253 is predicted to form hydrophobic contacts that bridge the CH domains. The decreased stability of the mutant indicates that these bridging interactions are probably disrupted, suggesting that the high F-actin binding affinity of the mutant is due to opening of the CH domain interface. These results support a fundamental role for leucine 253 in regulating opening of the CH domain interface and binding of the ABD to F-actin. This study indicates that high-affinity actin binding of L253P β-III-spectrin is a likely driver of neurodegeneration.

Spinocerebellar ataxia type 5 (SCA5) is a human neurodegenerative disorder that causes disability through loss of coordinated movement of extremities, gait ataxia, slurred speech and abnormal eye movements^1^. Average age of onset is the third decade of life, with neurodegeneration typically restricted to the cerebellum^2^,^3^,^4^,^5^,^6^,^7^. The disease is autosomal dominant and stems from mutations in the *SPTBN2* gene that encodes the protein β-III-spectrin^8^. β-III-spectrin is expressed predominantly in the brain and is enriched in cerebellar Purkinje cells^9^,^10^. An essential role of β-III-spectrin for Purkinje cells was demonstrated by β-III-spectrin null mice that show ataxic phenotypes and gross degeneration of Purkinje cell dendritic arbors^10^,^11^,^12^. The functional unit of β-III-spectrin is considered to be a tetrameric complex composed of two β-spectrin subunits and two α-II-spectrin subunits. The spectrin tetramer binds to short 37 nm F-actin filaments^13^ to form a cytoskeletal structure beneath the plasma membrane that confers mechanical strength to the membrane and organization of membrane proteins^14^. In addition, β-III-spectrin participates in endomembrane trafficking through its interaction with the actin related protein, ARP1^15^,^16^. ARP1 likewise forms a 37 nm filament^17^ and is a component of the dynactin complex that facilitates transport mediated by microtubule motors.

β-III-spectrin spans 2390 amino acids and consists of an amino-terminal actin binding domain (ABD), a central region containing seventeen spectrin repeat domains, and a carboxy-terminal pleckstrin homology domain. Six distinct SCA5 mutations have been reported in literature. Five of these mutations, E532_M544del^8^, L629_R634del.InsW^8^, R480W^5^, T472M^6^ and E870del^7^ are in spectrin repeat domains. The sixth mutation results in substitution of a proline for leucine 253 (L253P) in the ABD^8^. Recently, a mouse model was reported in which β-III-spectrin carrying the E532_M544del mutation is expressed specifically in Purkinje cells^18^. This model demonstrated that mutant β-III-spectrin expression in Purkinje cells is sufficient to induce ataxic and cerebellar degeneration characteristic of SCA5 pathogenesis, and points to a Purkinje cell deficit as the cellular mechanism underlying SCA5 pathogenesis. However, the molecular mechanism by which the E532_M544del mutation, or the other spectrin repeat domain mutations, causes neurotoxicity has not been established.

The position of the L253P mutation suggests that it causes pathogenesis by disrupting the function of the ABD to bind actin or ARP1. To date, no study has examined the effect of the L253P mutation on the interaction of β-III-spectrin with actin. In this current work, we report the results of rigorous biochemical analyses to shed light on the effect of the L253P mutation on the structure of the ABD and its function to bind actin.

## Results

**Figure 1:**
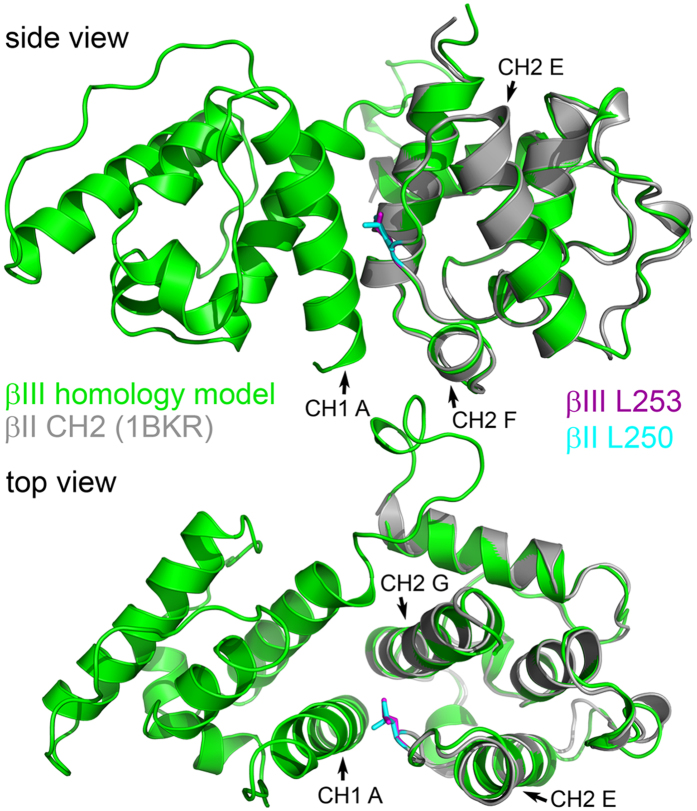
Leucine 253 is located in the E-F loop of the β-III-spectrin CH2 domain. A structural homology model generated for the β-III-spectrin ABD (CH1 and CH2 domains, amino acids 56 to 284) is shown in green. The side-chain of β-III-spectrin leucine 253 is colored magenta. The crystal structure of the β-II-spectrin CH2 domain (PDB ID: 1BKR) is shown in grey and is aligned with the β-III-spectrin homology model. β-II-spectrin leucine 250 is the equivalent residue of β-III-spectrin leucine 253. The side-chain of β-II-spectrin leucine 250 is colored cyan. β-III-spectrin leucine 253 is in a loop connecting alpha-helices E and F of the CH2 domain. The side-chain of β-III-spectrin leucine 253 is in close position to CH2 domain alpha-helix G and CH1 domain alpha-helix A.

The position of the spinocerebellar ataxia type 5 (SCA5) L253P mutation in the actin-binding domain (ABD) suggests that it confers neurotoxic properties to β-III-spectrin by disrupting the structure and/or function of the ABD. The ABD consists of two calponin homology (CH) domains in tandem, with leucine 253 located in the second calponin homology domain (CH2). To gain a detailed understanding of the positon of leucine 253 within the β-III-spectrin ABD, we examined an available crystal structure of the CH2 domain of the homolog β-II-spectrin^19^, together with a structural homology model of the β-III-spectrin ABD generated using the i-Tasser server^20^. The β-III-spectrin and β-II-spectrin CH2 domains are 87% identical in amino acid sequence. Figure 1 shows an overlay of the β-II-spectrin CH2 domain crystal structure with the β-III-spectrin ABD structural homology model. Similar to the β-II-spectrin CH2 domain crystal structure, the homology model shows that the β-III-spectrin CH2 domain consists of seven major alpha-helices (alpha-helices “A” to “G”). The alpha-helices of the β-II-spectrin and β-III-spectrin CH2 domains show excellent alignment, supporting the accuracy of the β-III-spectrin homology model. β-III-spectrin residue leucine 253, like the equivalent residue in β-II-spectrin, leucine 250, is located in the middle of a loop connecting alpha-helices E and F. The position of leucine 253 in a loop suggests that substitution of a proline residue, which has restricted backbone geometry not favorable for alpha-helix formation^21^, may be accommodated at this site without disrupting the surrounding alpha-helical structures.

**Figure 2:**
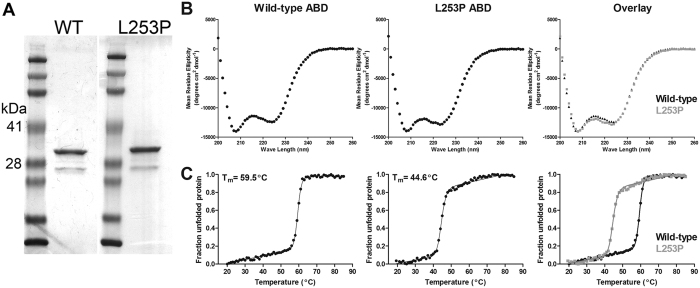
The L253P ABD has intact secondary structures but decreased thermal stability. (**A**) A Coomassie blue stained gel containing 2 μg of purified wild-type (WT) or L253P ABD protein resolved by SDS-PAGE. The β-III-spectrin ABD (amino acids 1–284) has a predicted molecular weight of 32.8 kDa and runs between the 28 and 41 kDa protein standards. A minor protein contaminant runs below the 28 kDa protein standard. (**B**) Circular dichroism spectra between 200 and 260 nm for the wild-type and L253P ABD proteins show pronounced alpha-helical profiles. (**C**) Circular dichroism melting curve analyses at 222 nm for wild-type and L253P ABD proteins show sharp, cooperative unfolding transitions. The L253P ABD has a melting temperature (Tm) that is 15 °C lower than wild-type.

To experimentally assess the effect of the L253P mutation on the structure and function of the β-III-spectrin ABD, the wild-type and mutant ABD proteins (amino acids 1–284) were bacterially expressed and purified, Fig. 2A. Circular dichroism (CD) spectroscopy was performed to assess secondary structure of the purified proteins. As expected, the CD spectrum for the wild-type ABD at 25 °C shows a pronounced alpha-helical profile, Fig. 2B. The CD spectrum for the mutant ABD also shows an alpha-helical profile that nearly overlaps the CD spectrum of the wild-type ABD. Thus the L253P mutation does not cause a substantial change in secondary structure. CD spectroscopy was further employed to assess the thermal stability of the ABD proteins. The wild-type ABD melts in a cooperative, two-state transition with a Tm of 59.5 °C, Fig. 2C. Similarly, the mutant ABD protein melts cooperatively, a further indication that the mutant protein has a well-folded state. However, the melting curve for the mutant ABD has a Tm of 44.6 °C, nearly 15 °C lower than the Tm of the wild-type ABD. Altogether these data demonstrate that the mutant ABD can attain a well-folded state similar to that of the wild-type protein, but the overall decreased thermal stability caused by the L253P substitution may cause β-III-spectrin to be prone to denaturation in mammalian cells.

**Figure 3:**
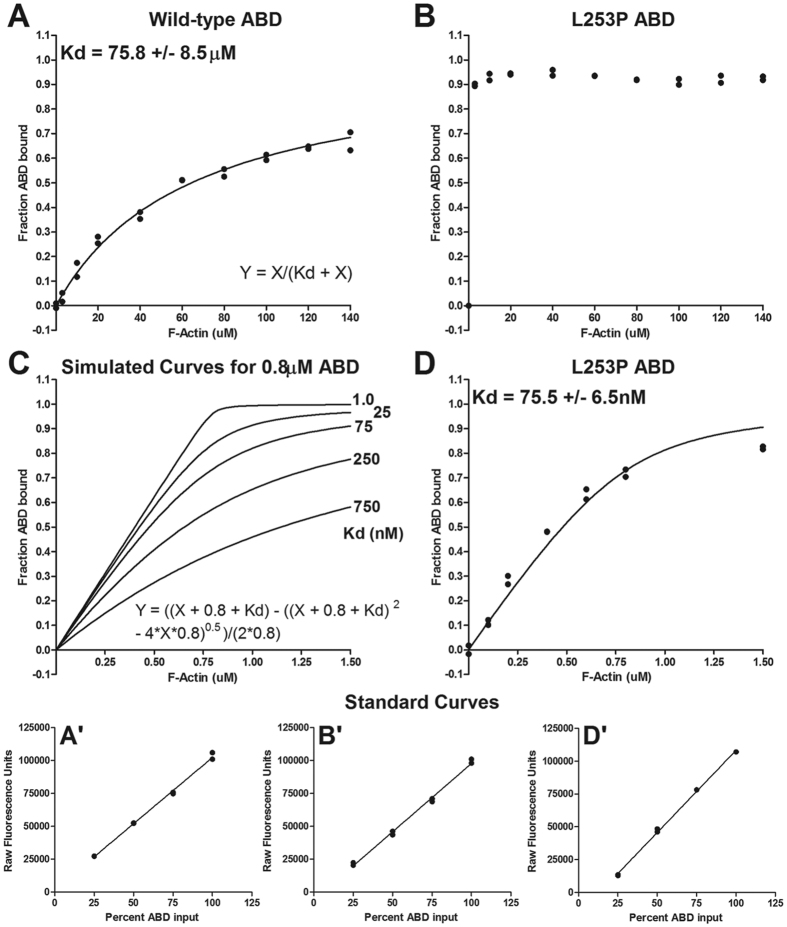
The L253P ABD binds F-actin with high affinity. (**A**) Results of an individual co-sedimentation assay performed using 2 μM wild-type β-III-spectrin ABD. The wild-type ABD has a Kd of 75.8 μM (average +/− standard deviation). This is the average Kd from six co-sedimentation assays using two ABD protein preps and four different F-actin preps. Curve fitting was performed using the equation given in this panel. (**B**) Results of an individual co-sedimentation assay performed using 2 μM L253P ABD and same range of F-actin concentrations as used for wild-type. Binding of the L253P ABD under these conditions was tested in three co-sedimentation assays with similar results. (**C**) Simulated curves using the alternative equation given in this panel showed that a sub-micromolar Kd estimate for the L253P ABD could be attained using 0.8 μM L253P ABD protein and low concentrations of F-actin. (**D**) Results of an individual co-sedimentation assay showing the binding of 0.8 μM L253P ABD to low concentrations of phalloidin stabilized F-actin. Data were fit to the equation given in panel (**C**) The Kd is the average Kd from three co-sedimentation assays using L253P ABD purified for a second time following the initial binding assays in panel **B**. **A**’,**B**’ and **D**’ are standard curves used to convert Coomassie blue fluorescence intensities to amount wild-type or L253P ABD protein for the binding data shown in panels (**A,B,D**) respectively.

To determine whether the L253P mutation affects the function of the ABD to bind F-actin, co-sedimentation assays were performed. The wild-type β-III-spectrin ABD bound F-actin with an average Kd of 75.8 μM, Fig. 3A. This Kd is ~3-fold higher than the 20 μM Kd reported for the homologous β-II-spectrin ABD contained in the equivalent amino-terminal sequence (1–281)^19^, or the 26 μM Kd reported for the β-II-spectrin ABD contained in a slightly extended amino acid sequence (1–313)^22^. Overall these data are consistent with a low-affinity interaction between F-actin and the ABD of β-spectrin. The initial co-sedimentation assays for the mutant ABD were performed using the same range of F-actin as for wild-type. Surprisingly, at the lowest concentration of F-actin (3 μM), ~90% of the 2 uM total mutant ABD protein was bound, Fig. 3B. At these same concentrations, less than ~5% of the wild-type ABD was bound to F-actin. This indicates that the mutant ABD binds F-actin with much higher affinity than wild-type. To gain an estimate of the Kd for the mutant ABD, co-sedimentations assays were performed using lower concentrations of F-actin and ABD, and alternative curve fitting, Fig. 3C,D. Under these reaction conditions the binding of the mutant ABD to F-actin is dose-responsive and fits a binding curve with an average Kd of 75.5 nM. This striking 1000-fold decrease in Kd suggests that the L253P mutation disrupts an essential mechanism in the ABD that is inhibitory to binding F-actin.

**Figure 4:**
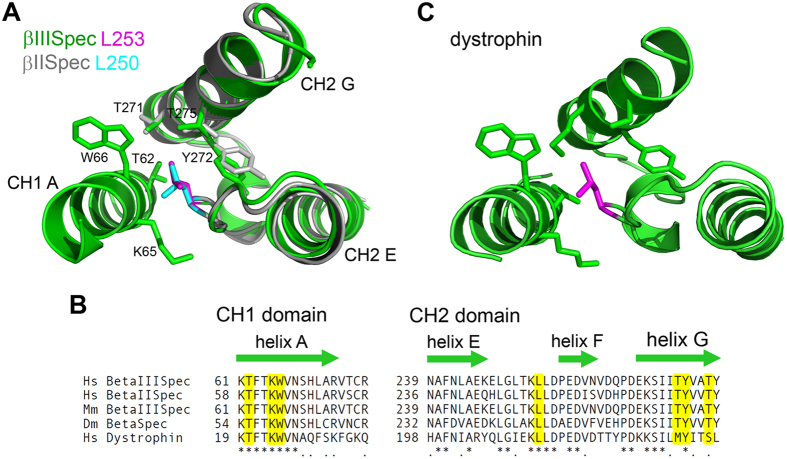
Leucine 253 side-chain hydrophobic interactions bridge the CH1-CH2 domain interface. (**A**) β-III-spectrin CH1 domain alpha-helix A and CH2 domain alpha-helices **E, F** and **G** (green) are shown aligned with β-II-spectrin CH2 domain crystal structure alpha-helices **E,F** and **G** (grey). The side chain of β-III-spectrin leucine 253 (magenta) or β-II-spectrin leucine 250 (cyan) is oriented towards CH1 domain alpha-helix A The β-III-spectrin leucine 253 side-chain is predicted to form hydrophobic interactions with threonine 62, lysine 65 and tryptophan 66 of CH1 domain alpha-helix A and with threonine 271, tyrosine 272 and threonine 275 of CH2 domain alpha-helix G. (**B**) ClustalW alignment showing that human (*Homo sapiens*, Hs) β-III-spectrin leucine 253 and its predicted hydrophobic contacts are conserved in human β-II-spectrin, mouse (*Mus musculus*, Mm) β-III-spectrin, fly (*Drosophila melanogaster*, Dm) β-spectrin and in the homologous ABD of human dystrophin. (**C**) The dystrophin residue equivalent of β-III-spectrin leucine 253, leucine 212 (magenta), is inserted into a nearly identical hydrophobic pocket at the CH domain interface in the crystal structure of the dystrophin amino-terminal ABD (PDB ID: 1DXX), a template structure used in generation of the β-III-spectrin homology model.

To gain insight into a possible F-actin binding regulatory mechanism mediated by leucine 253, we returned to the β-III-spectrin ABD structural homology model, which shows that the side-chain of leucine 253 is oriented towards the CH1 domain, Fig. 1. The leucine 253 side-chain is closely positioned to alpha-helix G in the CH2 domain and alpha-helix A in the CH1 domain. To determine potential leucine 253 hydrophobic contacts in these alpha-helices, side-chain aliphatic carbons within 4 angstroms, a typical aliphatic-aliphatic or aliphatic-aryl non-covalent bond distance^23^, were identified. In CH2 domain alpha-helix G, the side-chain of leucine 253 is predicted to form hydrophobic contacts with the side-chain methyl groups of threonine 271 and 275, and with the aromatic ring of tyrosine 272, Fig. 4A. In CH1 domain alpha-helix A, the predicted contacts are threonine 62, lysine 65 and tryptophan 66. In the case of lysine 65, leucine 253 contacts the aliphatic neck of the lysine side-chain. This lysine hydrophobic interaction is probably promoted by a neutralizing hydrogen bond^24^ formed between the lysine side-chain amine group and the backbone carbonyl oxygen of CH2 domain residue threonine 251. These atomic interactions point toward an important function of leucine 253 to bridge the CH1 and CH2 domains. Leucine 253 and the interacting residues are conserved in β-spectrin proteins across species, and in spectrin-related proteins including dystrophin, Fig. 4B,C. This suggests that bridging of the CH domains by leucine 253 or its equivalent is fundamental to the function of tandem CH domain ABDs found in β-spectrin and spectrin-related proteins. The effect of the L253P substitution to decrease thermal stability of the ABD but not cause widespread changes in secondary structure (Fig. 2) supports a local destabilization of the leucine 253 hydrophobic pocket at the CH1-CH2 domain interface, suggesting that the high F-actin binding affinity of the L253P mutant is due to opening of the CH1-CH2 domain interface.

## Discussion

This work identifies high-affinity F-actin binding as a likely molecular mechanism by which the human spinocerebellar ataxia type 5 (SCA5) L253P mutation confers neurotoxic properties to β-III-spectrin. The low actin-binding affinity (Kd = 75 μM) of the wild-type β-III-spectrin ABD suggests that normal membrane function requires a dynamic spectrin cytoskeleton in which spectrin-F-actin linkages form and dissociate. As postulated previously^14^, the low binding affinity of β-spectrin proteins for F-actin may allow factors such as protein 4.1^25^ and adducin^26^, which increase affinity of β-spectrin-F-actin linkages, to provide necessary localized regulation of the spectrin cytoskeleton in cells. The protein 4.1 isoform, 4.1B, is clearly expressed in Purkinje cells^27^, suggesting this protein is an important player in regulation of the spectrin cytoskeleton in the target cell of SCA5 pathogenesis. We predict that the high F-actin binding affinity (Kd = 75 nM) of the L253P β-III-spectrin ABD causes neurotoxicity by hindering regulation of the spectrin cytoskeleton and interfering with membrane plasticity required for proper Purkinje cell function.

L253P mutant β-III-spectrin may disrupt multiple cellular functions based on the broad subcellular localization of β-III-spectrin in the Purkinje cell soma, dendritic shafts and dendritic spines^9^,^12^. F-actin is intensely concentrated in Purkinje cell dendritic spines^28^,^29^, suggesting that β-III-spectrin-F-actin interactions are particularly important in this membrane compartment. Indeed β-III-spectrin is essential to spine function, as null mice show grossly reduced spine densities in Purkinje cells^12^, and Purkinje cells in mice expressing E532_M544del mutant β-III-spectrin show decreased dendritic spine localization of metabotropic glutamate receptor 1 alpha (mGluR1α) and reduced mGluR1α signaling^18^. Similar to the localization of β-III-spectrin to Purkinje cell dendritic spines, the β-I-spectrin isoform is localized to dendritic spines of hippocampal pyramidal cells^30^. When expressed in hippocampal pyramidal neurons, the ABD of β-I-spectrin localizes to dendritic spines and alters spine morphology and synaptic function^31^. Further, the β-I-spectrin ABD inhibits the ability of spines to alter morphology in response to an F-actin depolymerizing agent, latrunculin-B, suggesting that the deleterious effects of the β-I-spectrin ABD to spine function is due to stabilization of F-actin. It is possible that the increased F-actin binding affinity of L253P β-III-spectrin likewise interferes with F-actin dynamics and disrupts dendritic spine function in Purkinje cells.

In addition to the function of β-III-spectrin ABD to mediate the interaction of β-III-spectrin with F-actin, the ABD also links β-III-spectrin to the actin related protein, ARP1, which functions in the dynactin protein complex to facilitate microtubule transport. Current evidence suggests that the β-III-spectrin ABD binds to actin by a distinct mechanism compared to ARP1; F-actin failed to compete with ARP1 for binding the β-III-spectrin ABD^15^. We previously demonstrated that the L253P mutation, introduced in the *Drosophila* homolog β-spectrin, interferes with the motility of individual synaptic vesicles in *Drosophila* motor neurons^32^, suggesting a defect in β-spectrin binding to ARP1 on the surface of vesicles. This suggests that the effect of the L253P mutation may be pleiotropic, conferring neurotoxicity by disrupting both conventional actin and ARP1 mediated cell functions. A bimolecular fluorescence complementation assay suggested that the L253P mutation decreases the interaction of β-III-spectrin with ARP1^33^. Thus it will be interesting, in a future line of investigation using *in vitro* and *in vivo* studies with native forms of proteins, to confirm and quantitatively assess the effect of the L253P mutation on β-III-spectrin binding to ARP1. Irrespective of the interaction of β-III-spectrin with ARP1, the L253P mutation results in a significant increase in the affinity of β-III-spectrin for conventional actin and predicts that disrupted actin-mediated functions underlie SCA5 pathogenesis.

**Figure 5:**
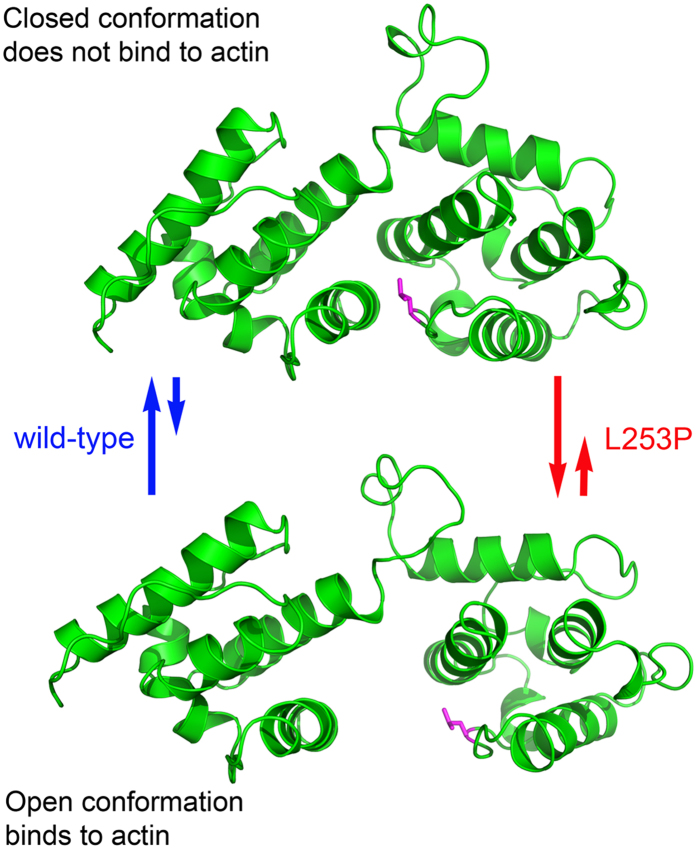
Model: Closed and open conformations control binding of the β-III-spectrin ABD to F-actin. In the closed conformation (top structure, represented by the β-III-spectrin structural homology model) the two CH domains closely associate and this interaction is promoted by leucine 253 and its CH1 domain hydrophobic contacts. In the open conformation (bottom structure, CH1 and CH2 domains of the structural homology model were arbitrarily separated), leucine 253 no longer contacts the CH1 domain and the two CH domains are spatially separated. The closed conformation does not bind actin while the open state favors interaction with actin. The closed and open conformations may exist in equilibrium or the open conformation may correspond to a transition state that occurs in the F-actin binding reaction. The L253P mutation causes the ABD to adopt the open conformation by disrupting CH1-CH2 domain hydrophobic interactions normally mediated by leucine 253.

Finally, this study sheds greater light on the basic biology of β-spectrin proteins by pointing to a fundamental role for the evolutionarily conserved residue leucine 253 in regulation of F-actin binding. Our data support a model, as illustrated in cartoon format in Fig. 5, in which the wild-type ABD can adopt “closed” and “open” conformations. In the closed conformation, the two CH domains closely associate and this interaction is promoted by leucine 253 and its CH1 domain hydrophobic contacts. In the open conformation, leucine 253 no longer contacts the CH1 domain and the two CH domains are spatially separated. The closed conformation does not bind actin while the open state favors interaction with actin. The closed and open conformations may exist in equilibrium, or the open conformation may correspond to a high energy transition state adopted by the ABD in the F-actin binding reaction. Future FRET or DEER studies, similar to those that demonstrated an actin-induced opening of the homologous ABD from utrophin^34^, will clarify the mechanism. Regardless, the best explanation for the high-affinity actin binding caused by the L253P mutation is that it causes the ABD to adopt an open conformation by disrupting CH1-CH2 domain hydrophobic interactions normally mediated by leucine 253.

## Materials and Methods

### Protein Purification

The coding sequence for the wild-type or L253P human β-III-spectrin ABD (amino acids 1–284) was sub-cloned into pET-30a(+) (Novagen) such that the native ABD sequence without a foreign epitope tag can be expressed in bacteria. Briefly, the ABD coding sequences were PCR amplified from pUASp-hSPWT or pUASp-hSPGM templates^32^ using the forward oligo GGAATTCCATATGAGCAGCACGCTGTCACCC and reverse oligo ACCCAAGCTTCTACTTCATCTTGGAGAAGTAATGGTAGTAAG. The PCR product was digested with Nde1 and HindIII and sub-cloned into pET-30a(+) digested with the same enzymes. The final constructs pET-30a-ABD WT and pET-30a-ABD L253P were sequenced verified and then transformed into *Escherichia coli* BL21(DE3) (Novagen). Transformed bacteria were incubated with rotation at 37 °C in a flask containing 1L LB media and 50 μg/mL kanamycin until an absorbance of 0.4 at 550 nm was reached. Then the flask was placed in ice for 15 min before addition of IPTG to 0.33 mM final. The flask was then incubated with rotation for 4 h at 22 °C. We found that expression at 22 °C increased the amount of soluble L253P ABD protein. Bacteria were harvested at 5,000 x *g* and pellet stored at −20 °C. Bacteria were lysed with lysozyme (Sigma) for 1 h at 4 °C in buffer containing 50 mM Tris pH 7.5, 1 mM EDTA, 25% sucrose with protease inhibitors (Complete Protease Inhibitor tablet, EDTA-free, Roche), followed by a freeze-thaw cycle using an isopropanol-dry ice bath. Then MgCl_2_ to 10 mM final and DNase1 (Roche) to 7.5 U/mL final were added and lysate incubated for 1 h at 4 °C. Lysate was clarified at 40,000 x *g* at 4 °C for 30 min. Supernatant was collected and passed through a 0.2 μM syringe filter before loading onto an anion exchange column (HiTrap Q XL, GE) equilibrated in buffer containing 20 mM Tris pH 7.5, 1 mM EGTA and 1 mM DTT at 4 °C. Proteins were eluted from the column using a linear gradient of NaCl from 0 to 500 mM. SDS-PAGE and Coomassie blue staining was performed to identify fractions enriched with the ABD. The fractions were pooled and concentrated (Amicon Ultra-15 Centrifugal Filter, 10 K MWCO) to 5 mL before loading onto a gel filtration column (Sephadex S200, GE) equilibrated with buffer containing 10 mM Tris pH 7.5, 150 mM NaCl, 2 mM MgCl_2_ and 1 mM DTT at 4 °C. Elution fractions were analyzed by SDS-PAGE and Coomassie blue staining and fractions enriched with the ABD pooled and concentrated (Amicon Ultra-15 Centrifugal Filter, 10 K MWCO).

### Circular dichroism measurements

Prior to analyses, purified ABD proteins were clarified at 100,000 x *g* at 4 °C for 30 min. The clarified ABD protein concentrations were determined by Bradford assay (Biorad) and ranged from 217 ng/μL to 197 ng/μL. CD spectra were acquired in a Jasco J-815 Spectropolarimeter equipped with a Peltier temperature controller. Immediately before analysis, the instrument was baseline-corrected using ABD protein buffer (10 mM Tris pH 7.5, 150 mM NaCl, 2 mM MgCl_2_ , 1 mM DTT). For secondary structure analyses, CD spectra were measured from 200 nm and 260 nm at 25 °C. Immediately following, the thermal unfolding of the ABD protein sample was analyzed by recording CD spectra at 222 nm over the temperature range of 20 °C–85 °C. Non-linear regression analysis was performed in Prism 5 (GraphPad Software, Inc.) to determine the melting temperature using an equation for a two state transition, as reported previously^35^.

### F-actin co-sedimentation assays

F-actin was prepared from rabbit skeletal muscle^36^. The purified ABD proteins were clarified at 100,000 x *g* at 4 °C for 30 min just prior to setting up binding assays. Concentrations of F-actin and clarified ABD were determined by Bradford assay (Biorad). Binding assays were performed in F-buffer containing 10 mM Tris pH 7.5, 150 mM NaCl, 0.5 mM ATP, 2 mM MgCl_2_, and 1 mM DTT. In Fig. 3A,B, binding assays contained 2 μM ABD protein and F-actin ranging from 0 to 140 μM in a total volume of 60 μL. Binding reactions were allowed to reach equilibrium at room temperature (23–24 °C) for 30 min and then F-actin pelleted by centrifugation at 100,000 x *g* at 25 °C for 30 min. The amount of unbound ABD was sampled by combining 40 μL of binding assay supernatant with Laemmli sample buffer and performing SDS-PAGE followed by Coomassie blue staining. After destaining, gels were scanned for Coomassie blue fluorescence using the 700 nm channel in an Odyssey Imager (LI-COR Biosciences). The fluorescence intensities of the ABD protein bands were quantified in Image Studio Lite v4.0 software (LI-COR Biosciences). Individual ABD band fluorescence intensities were converted to amount ABD protein. To perform this conversion a standard curve was generated by linear regression in Prism 5 (GraphPad Software, Inc.) using ABD Coomassie blue fluorescence intensity values attained from a SDS-PAGE gel loaded with varying amounts of ABD in F-buffer. To determine dissociation constant (Kd) values, data were fit by non-linear regression in Prism 5 software to the equation *Y* = *X/(Kd* + *X)*, where Y equals fraction ABD bound and X equals free F-actin concentration^37^. The binding assay in Fig. 3D was performed as above but with following modifications: F-actin, stabilized using phalloidin in a 1:1 molar ratio, was varied from 0 to 1.5 μM. The ABD concentration was held constant at 0.8 μM. This 0.8 μM ABD concentration was estimated by multiplying the concentration of the purified ABD protein solution determined by Bradford assay by a correction factor to account for a minor low molecular weight protein contaminant (see Fig. 2A). The correction factor is based on protein band fluorescence intensities measured in Coomassie blue stained SDS-PAGE gels and equaled the quotient of the fluorescence intensity of the ABD protein divided by the sum of fluorescence intensities of the ABD protein and the contaminating protein. Non-linear regression analysis was performed in Prism 5 to fit binding data to the equation *Y* = *((X* + *0.8* + *Kd) – ((X* + *0.8* + *Kd)*^2^ – 4*X*0.8)^0.5^)/(2*0.8), where Y = fraction ABD bound, X equals total F-actin concentration, and 0.8 equals total ABD concentration in μM units^37^.

### Structural modeling

The β-III-spectrin ABD structural homology model was generated by submitting the β-III-spectrin amino acid sequence 56–284 to the i-Tasser server^20^. The PDB IDs of the top template structures used by the server are 1DXX (dystrophin), 2EYI (alpha-actinin 1), 1WKU (alpha-actinin 3), 3F7P (plectin 1), 1SSJ (alpha-actinin) and 1TJT (alpha-actinin 3). The C-score for the homology model is 1.21. Alignment of the β-III-spectrin structural homology model to the crystal structure of the β-II-spectrin CH2 domain was performed in PyMOL v1.30 (Schrodinger, LLC). Identification of side-chain aliphatic carbons within 4 angstroms of the leucine 253 side-chain was also performed in PyMOL v1.30. The open conformation of the β-III-spectrin ABD in Fig. 5 was generated by modifying the homology model in Discovery Studio 4.1 Visualizer (Accelrys).

## Additional Information

**How to cite this article**: Avery, A. W. *et al*. A human b-III-spectrin spinocerebellar ataxia type 5 mutation causes high-affinity F-actin binding. *Sci. Rep.* **6**, 21375; doi: 10.1038/srep21375 (2016).
